# The Association Between Dental Anxiety And Psychiatric Disorders And Symptoms: A Systematic Review

**DOI:** 10.2174/1745017901814010207

**Published:** 2018-08-31

**Authors:** Harri Halonen, Jenna Nissinen, Heli Lehtiniemi, Tuula Salo, Pirkko Riipinen, Jouko Miettunen

**Affiliations:** 1 Oulu University Hospital, Department of Psychiatry, 90230 Oulu, Finland; 2 Center for Life Course Health Research, University of Oulu, 90230 Oulu, Finland; 3 Department of Diagnostics and Oral Medicine, Institute of Dentistry, University of Oulu, 90230 Oulu, Finland; 4 Institute of Clinical Medicine, Department of Psychiatry, University of Oulu, 90230 Oulu, Finland

**Keywords:** Dental anxiety, Comorbidity, Anxiety disorders, Specific phobia, Psychiatric symptoms, Psychiatric disorders

## Abstract

**Background:**

A growing amount of evidence suggests that dental anxiety is associated with other psychiatric disorders and symptoms. A systematic review was conducted to critically evaluate the studies of comorbidity of dental anxiety with other specific phobias and other Axis I psychiatric disorders.

**Objective:**

The aim of the review was to explore how dental anxiety is associated with other psychiatric disorders and to estimate the level of comorbid symptoms in dental anxiety patients.

**Methods:**

The review was conducted and reported in accordance with the MOOSE statement. Data sources included PubMed, PsycInfo, Web of Science and Scopus.

**Results:**

The search produced 631 hits, of which 16 unique records fulfilled the inclusion criteria. The number of eligible papers was low. Study populations were heterogeneous including 6,486 participants, and a total of 25 tests and in few cases clinical interviews were used in the evaluation processes. The results enhanced the idea about the comorbidity between dental anxiety and other psychiatric disorders. The effect was found strong in several studies.

**Conclusion:**

Patients with a high level of dental anxiety are more prone to have a high level of comorbid phobias, depression, mood disorders and other psychiatric disorders and symptoms.

## 1 INTRODUCTION

Dental anxiety is a phenomenon that is seen almost every day in dental offices, and it can be a major challenge for both patient and a dental care provider. Between roughly one patient out of eight and one patient out of six reports, the level of fear that can be problematic in dental treatment [1-8]. The level of experienced anxiety can vary from a tension that a patient can cope with to absolute avoidance behaviour. There is a great variation of tests used for measuring dental anxiety which may cause some difficulties in interpretation of the prevalence [5]. However, it can be stated that the fear of dental treatment is quite common among patients. Roughly two-thirds of the patients are women [2, 6].

Fear of dental treatment is described in both ICD-10 classification and Diagnostic and Statistical Manual of Mental Disorders, DSM-IV, in specific (isolated) phobias, a subgroup of anxiety disorders [9, 10]. According to criteria, dental anxiety (phobia) is a phobia restricted to highly specific situations, although the contact with the phobic situation can evoke panic as in the cases of agoraphobia or social phobia [9]. The person recognises that the fear is excessive or unreasonable and the phobic situation is avoided or else is endured with intense anxiety. Also, the avoidance or distress in the feared situation interferes significantly with the person’s normal routine, occupational functioning or social relationships [10].

Patients with dental fear also show a number of other comorbid phobias: agoraphobia, social phobia and other specific phobias - fear of heights, enclosed spaces, animals, blood or natural phenomena [2, 3, 11-13]. Further, dental fear is known to have a positive correlation with depression and mood disorders [2, 3, 12], alcohol dependence [4] and substance abuse [12], and a variety of different psychiatric disorders [2, 3, 11] with a tendency that the higher the level of dental anxiety, the higher the presence of other comorbid phobias and disorders [2, 3]. The comorbidity between dental anxiety and different phobias and disorders has been found in patients of dental clinics [12, 13], in a birth cohort study [2, 3] and in a large population collected from volunteers [11].

Other specific phobias (besides dental phobia) can precede anxiety disorders or depression [14-16]. Also, in general, anxiety-related disorders have high comorbidity with depression and mood disorders, or alcohol/substance use disorder, and vice versa [17-19]. Some clinical symptoms of anxiety and depression can overlap [20], suggesting a common neurobiological basis of the disorders. However, there has been no neurobiological research on these conditions in (primary) dentally fearful patients.

Traditionally, dental anxiety is seen as a separate, independent fear caused by bad dental experiences. The studies about “separate” fear are well-presented in literature, while a number of studies about the comorbidity of dental anxiety with psychiatric disorders are quite low. No previous systematic review of comorbidity between these exists. In this study, we systematically collected previous studies of dental anxiety and comorbid (specific) phobias and other psychiatric symptoms and disorders. The aims of this systematic review were to i) explore how dental anxiety is associated with other psychiatric disorders in adult dental anxiety patients ii) estimate the difference between comorbid diagnoses and /or the severity of symptoms between the anxiety patients and controls iii) estimate associations of dental anxiety symptoms with comorbid symptoms in any adult patients and any adult populations.

Due to various terms used to describe fear over dental treatment in previous literature, we now use one single term, dental anxiety to mean the subject of interest regardless of the term used in the original article.

## 2 MATERIAL AND METHODS

This present study protocol followed the MOOSE [21] guidelines (Stroup *et al.* 2000).

### 2.1 Search Strategy

PubMed, PsycInfo, Web of Science and Scopus databases were screened using search strategy: “(“dental anxiety” OR “dental phobia” OR “dental fear”) AND (“generalized anxiety disorder” OR “anxiety disorder” OR “panicdisorder” OR “agoraphobia” OR “social phobia” OR “specific phobia” OR “simple phobia” OR “phobia” OR “obsessive-compulsive disorder” OR“post-traumatic stress disorder” OR “mood disorder” OR “depression” OR “dysthymic disorder” OR “dysthymia” OR “bipolar disorder” OR“substance abuse” OR “substance dependence” OR “substance use disorder” OR “alcohol abuse” OR “alcohol dependence” OR “alcohol use disorder” OR “psychosis” OR “schizophrenia”)”. The last search was run on 25^th^ of September 2015, except PsycInfo, of which the last run was made 28 Feb 2014 (due to availability). The search produced 1,132 results (PubMed 311, PsycINFO 70, Web of Science 278 and Scopus 473 hits). After duplications were removed, 631 unique publications were identified.

First, the material was screened (HH and JN) on the basis of abstract, and if not available, a full-text article was obtained. Non-English articles were translated if necessary.

**Fig. (1):**
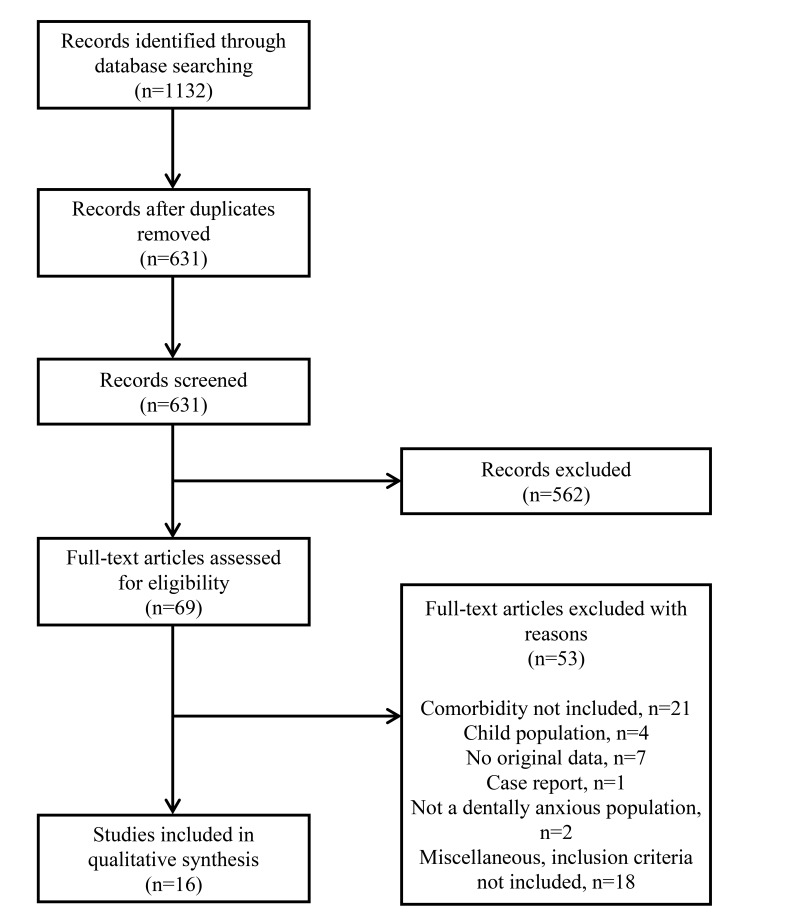
Flow chart.

Records thought to fulfill the inclusion criteria were labelled “yes” or “maybe” and full-text articles were obtained. A group of four unblind reviewers (authors) judged the material by using the 10-item checklist, scoring each from 0-1 points (see **Appendix 1**) modified from a checklist created for the assessment of randomised and non-randomised studies [22]. Disagreements on the exclusion/inclusion of the record were resolved by consensus. Reference lists of accepted articles were then hand-checked for additional studies fulfilling the inclusion criteria (see the flowchart in Fig. **1**).

### 2.2 Inclusion / Exclusion Criteria

The material was accepted for review if the original study included the examination of comorbidity of dental fear with other specific phobias or psychiatric disorders and symptoms.

The material was included if one or more of the following methods was used to evaluate dental anxiety: i) in general populations, the level of anxiety was measured by test or questionnaire, ii) if the study population consisted of (referred or self-referred) patients seeking treatment from a dental fear clinic, or iii) if the patients fulfilled the specific dental phobia criteria described in the ICD-10 [9] or DSM-III [23] / DSM-IV [10]. We decided to exclude material if dental anxiety was self-reported by single item or question, since narrative and subjective experiences can mean almost any level of fear, and there is no effort to objectively evaluate the level of dental fear.

In the same manner, we accepted the material if comorbid phobias and disorders were studied i) using a test, ii) a self-report questionnaire(s) or iii) a psychiatric clinical interview according to ICD-10 or DSM-III/DSM-IV criteria.

We decided to include the material if the study populations consisted only of adult patients or other individuals. The age limit was set at 18 years.

Case reports and treatment considerations were excluded from the review. We also excluded all studies in an effort to explain the origin of dental anxiety if the comorbidity was not examined in the original study.

In order to reduce publication bias, no limits were set to publication language or year.

### 2.3 Procedure

If the original study included both cases and controls, the difference between comorbid diagnoses and /or the severity of symptoms was compared between the groups. If only cases were included, we then evaluated how dental anxiety was associated with other symptoms and also estimated the level of comorbid diagnoses. In population-based studies, the association of the dental anxiety/phobia with other psychiatric diagnoses and symptoms was estimated.

The effect size estimates (Cohen’s d values, correlations or odds ratios) in each study were categorised as small (*), moderate (**) and large (***) effects, based on the cut-offs presented by Rosenthal [24]. For the cut-offs used, see Table **2**.

## 3 RESULTS

### 3.1 Material

The search produced 631 hits, of which, 16 unique articles fulfilled the inclusion criteria and were reviewed (see the flowchart in Fig. **1**). Finally, all of these were in English. The most common reasons for exclusion were that no tests/interviews were used or that the comorbidity was not examined in the article.

In the articles reviewed, five different structured tests (see **Appendix 2**) were used to measure dental anxiety; the Dental Anxiety Scale DAS [25], the Dental Beliefs Survey [26], the Dental Fear Survey [27], the Gatchel Fear Scale [28] and the Modified Dental Anxiety Scale MDAS [29]. A total of 20 tests were used to evaluate comorbid phobias and disorders. In three of the studies [12, 31, 33], clinical interviews were used.

### 3.2 Study Populations

The articles reviewed included a total of 6,486 adult patients, students, volunteers and other participants. Study populations were heterogeneous; patients were collected from birth cohort studies (two articles), dental fear clinics (eight articles), randomly selected volunteers (three articles) and three other populations; university students (one article), university employees population (one article) and patients scheduled for an appointment at university dental clinic (one article).

### 3.3 Dental Anxiety and Comorbid Phobias

Dental anxiety was significantly correlated with agoraphobia [2, 3, 11, 30] and social phobia [2, 3, 13, 31]. The association between dental anxiety and various specific (simple) phobias was found in seven studies of which the effect size was strong (***) in five: DeJongh [32] *et al.* 1998***, Hägglin [33] *et al.* 2001***, Locker [11] *et al.* 1997***, Locker [2, 3] *et al.* 2001, Moore [31] *et al.* 1995, Roy-Byrne [12] *et al.* 1994***, Tellez [13] 2015***. The results showed that the prevalence of co-existing simple phobia can be up to 45% [12]. The results were equal in patients of special dental fear clinics and with patients from birth cohort studies or selected randomly and then compared to the normative population.

### 3.4 Dental Anxiety and Comorbid Psychiatric Disorders and Symptoms

Results also had a similar degree of associations with comorbidity in different populations in studies of dental anxiety and comorbid psychiatric disorders Tables **1a** and **b**.

Dental anxiety had a significant positive correlation to depression and mood disorders in four population-based studies [2, 3, 33, 35] and in four studies of dentally fearful patients [12, 30, 36, 37].

The positive association between dental anxiety and (other) anxiety disorders was found in a total of nine studies, and, in five of them, the association was found to be strong (***): Aartman [30] *et al.* 1997***, DeJongh [32] *et al.* 1998***, Hakeberg [36] *et al.* 2001***, Halonen [34] *et al.* 2014***, Hägglin [33] *et al.* 2001, Kaakko [35] *et al*. 1998, Locker [2, 3] *et al.* 2001***, Pekkan [37] *et al*. 2011 and Roy-Byrne [12] *et al.* 1994 (for cut-offs used in effect size estimates, see Table **2**. Five of these were studies of dentally fearful patients. The association between dental anxiety and generalised anxiety disorder was found in one study [31].

Other significant comorbid findings included alcohol and substance use disorder [2, 3, 12], and, also other DSM-IV Axis I disorders [2, 3, 11, 30, 33].

## 4 DISCUSSION

### 4.1 Main Findings

The comorbid diagnoses and psychiatric symptoms were found equally in population-based studies and in studies with patients of dental fear clinics Table **2**.

Some main findings emerged, however. The most common comorbid finding with association with dental fear was anxiety-related disorders; a positive correlation was found in one birth cohort study, two randomly selected populations, one university student volunteer population, a total of four populations of special dental fear clinic patients and one population of patients seeking treatment at a university dental clinic.

The positive association of dental anxiety to depression and mood disorders was proven in eight different original studies (a total of three populations of a special dental fear clinic, one birth cohort study, two randomly selected populations, one university student volunteer population and one population of patients seeking treatment at a university dental clinic).

Agoraphobia and social phobia were found to be associated with dental anxiety in three original studies, and, other specific (simple) phobias in seven studies. Commonly, patients with dental anxiety had more than one additional specific phobia; most common simple phobias were blood-injection-injury-related phobias. In all phobia subtypes, the association was found both in population-based and dental fear clinic patients.

The gender difference was observed in seven articles. In all cases, women had more comorbidity between dental anxiety and general fears and phobias, depression and blood-injury fears than men [11, 30, 32, 34, 37-39].

Even though the type of representation of the results was scattered in the original articles, these findings were coherent in all studies, thus enhancing the idea of similarity between dental fear and these factors.

### 4.2 Methodological Discussion

In only three different studies [12, 31, 33] were clinical interviews used, while the others were based only on the use of different questionnaires, which brings some criticism to the interpretation of the results. However, the main findings were quite similar between these two methods; except generalized anxiety disorder, which was only found in a clinical interview-based study [31], and agoraphobia, found in three questionnaire-based studies [2, 3, 11, 30].

The tests used partly overlap: Some psychiatric and specific phobia questionnaires also include scales to measure dental fear. Most tests used in original papers are known to be commonly used in clinical practice.

The results also suggest that dental anxiety either increased or decreased in concert with the number of other fears [32], and, in a longitudinal study [33] during the follow-up drop-outs had a higher level of dental fear (Table **1a**).

Some findings in the screening process and in clinical evaluation also support the results. For example, the personality trait neuroticism, understood as incapability to cope with psychological stress, is associated with dental anxiety and its maintenance over time [33, 40, 41]. Neuroticism is well known to be connected with (general) anxiety disorders, depression and substance use disorders. Some of the latest findings suggest that genetic factors play at least some role in the development of fear [42, 43]. However, in order to limit the focus, we decided to exclude studies about personality traits, genetic factors and dental anxiety, since they best explain the origin, not comorbidity of dental fear.

The number of articles identified was relatively low when taking into account a large number of studies on dental anxiety (the term “dental anxiety” produced over 4,000 hits in PubMed). The idea of similarity with other fears and a patient’s role in the acquisition of fear are quite new, and so is the psychological testing in the research work. In the evaluation process, many original articles were excluded because of the methodology, despite careful study design and patient selection.

### 4.3 Strengths and Limitations

As far as we know, no previous systematic reviews of the comorbidity of dental anxiety to other psychiatric disorders and symptoms exist. The search strategy was very representative and thorough, including Axis I categories of mood disorders, anxiety disorders, substance-related and psychotic disorders. Although the number of articles was only a small minority of all search hits (about 2.5 percent), we believe that we have located all the relevant studies on the topic.

The tests used in the original articles and even the aims of different studies were very heterogeneous. Because of this heterogeneity in methods, we decided not to pool the results for a meta-analysis, although this left us a body of descriptive data for a systematic and critically review the articles. However, when possible, we estimated the magnitude of the effects.

This heterogeneity was also a benefit; when the same result based on an ICD-10 or DSM-IV classification is made by multiple tests, it will increase the reliability of the results, thus lowering the bias. In the same manner, the heterogeneity of the study populations gave a representative and large sample of dentally anxious individuals.

The *limitation* to the interpretation of our results is the question of how diagnoses were made and symptoms were assessed in the original papers. The diagnosis of (specific) dental phobia, as well as any other phobia, depression, or other disorder, is always made by a psychiatrist. A test, for example, for anxiety or depression inventory, can be used for research purposes, but it is not a clinical diagnosis. Only in a few of the papers reviewed were clinical interviews used.

The prevalence of anxiety disorders in large national epidemiologic surveys [44-46] can vary between 14.4 and 18.1%, while the prevalence of mood disorders can be up to 9.5% [45]. If a control group is used in the original study, as was the case in eight (half) of the reviewed articles, the level of this “natural” co-existing anxiety/mood disorders can be evaluated and distinguished from the disorders associated with dental fear. However, in the other half of the articles, the control group was not used in the study, so the results must be interpreted carefully.

Also, some of the original studies were cross-sectional and some had a longitudinal aspect, suggesting that there may be a trait (dental) anxiety and state (dental) anxiety involved. We were not able to study these separately from the articles included.

Although no limits were set to publication language or year, with only a few exceptions, all the records identified through database searching were in English. This may be due to language bias in the databases. The results were relatively robust across all the articles, but it is possible that there exists some publication bias. It is possible that studies have been conducted in which the association was not found to be significant, but such studies have not been published or made available anywhere.

## CONCLUSION

A high level of dental anxiety (dental fear) has a strong positive correlation with a high level of other comorbid phobias, depression, mood disorders and other psychiatric disorders and symptoms. However, on the basis of the articles reviewed, it cannot be concluded whether patients with dental anxiety are more prone to have a high level of other psychiatric disorders, or whether other psychiatric conditions are the primary disorder predisposing a given individual to the development of dental anxiety. This brings a challenge to clinical work; how should a dentist manage a patient with dental anxiety, or should a patient be advised to consult a psychologist or a psychiatrist? Further examinations are needed.

## Figures and Tables

**Table 1a:** Dental anxiety and comorbid phobias and psychiatric symptoms. Control groups were included in the original studies.

**Author**	**Study Population**	**Aim of the Study**	**Methods, Used Tests**	**Comorbid Phobias**	**Comorbid Psychiatric Symptoms**	**Comments**
Aartman [30] *et al*. 1997	Patients of Dental fear clinic, n=321. Study group was compared to a normative sample of the Dutch general population, n=1009.	To assess psychological characteristics of highly anxious patients	● SLC-90	Patients scored significantly higher in subscale “agoraphobia” than normative population (P<0.001, d=0.55 in men, P<0.001, d=0.53 in women)	Patients scored higher - anxiety (P=0.000, d=0.83 in men, P=0.000, d=0.84 in women) - depression (P=0.000, d=0.54 and P=0.000, d=0.72) - somatization (P=0.001, d=0.38, P=0.000, d=0.47) - cognitive-performance difficulty (P=0.005, d=0.33 and P=0.000, d=0.41) - interpersonal sensitivity and paranoid ideation (P=0.001, d=0.42, P=0.000, d=0.50)	Control group was not matched to study population. Dental anxiety patients scored higher in all subscales than Dutch normative population.
DeJongh [32] *et al*. 1998	● Sample A: 41 adult patients of dental fear clinic in Netherlands ● Sample B: 22 adult patients comparable to sample A ● Sample C: 173 patients (aged 11-84yrs, mean 33.6) of department of dental surgery	To determine the relationship between characteristics of dental fear and BII (blood-injection-injury) fears To explore the degree of overlap between dental phobia and BII phobia on the basis of DSM-IV criteria	● Sample A: - DAS - the Blood-injury Scale of the FQ-BI - SLC-90-R - a 22-item questionnaire constructed for the purpose of the present study - DQ ● Sample B: - DAS - Phobia Checklist ● Sample C: - Phobia Checklist^49^ - Questionnaires about fainting	● Dental anxiety (DAS) was significantly correlated to general trait anxiety (SLC-anxiety) (r=0.55, p<0.001) but not to BII-fear (r=16). ● 56.7% of the patients with dental phobia had at least one additional BII subtype-specific phobia (only 7.9% of non-dental phobic patients had additional phobia). ● They also had a significantly higher proportion of any BII phobias than non-dental phobics (p<0.0001): - blood 10% vs. 2.4 - injection 45.5 vs. 5.5 - injury 23.3 vs. 3.0		The authors concluded that, despite the co-occurrence of dental phobia and blood-injection-injury phobia, dental anxiety should be considered a specific, independent phobia within DSM-IV.
Halonen [34] *et al*. 2014	Young adult university students, Finland, n=880. Participants scoring ≥19 (out of max. 25 pts) were classified as dentally anxious, 12-18 pts mildly anxious and ≤11 pts not anxious	To assess the correlation between dental anxiety, general clinical anxiety and depression.	●MDAS ●BAI ●BDI		Dental anxiety was significantly correlated with general anxiety (p<0.001) and depression (p<0.001) in females. DA was significantly correlated to general anxiety in men (p<0.016).	
Hägglin [33] *et al*. 2001	A random female population in Gothenburg, Sweden, n=310. A longitudinal study from 1968 to 1992. Participants were grouped into low, high or extreme dental fear subgroups by the Phobia questionnaire.	To examine levels in dental anxiety in relation to other fears/phobias longitudinally	● The phobia self-referred questionnaire (number of phobias) ● **Psychiatric interview** (major depression DSM-III, psychiatric impairment) ● Self-reported anxiety level	● High dental fear at baseline was associated with a higher number of other phobias (mean 1.3 vs. 4.7, P<0.001)	High dental fear at baseline was associated with more psychiatric impairment (10.7% vs. 18.3%, P=0.003), social disability (1.7% vs. 10.8%, P<0.001) and anxiety (21.5% vs. 30%, P=0.007). Depression was higher in chronic group (*i.e.* highly dentally anxious at 1968 and 1992) than incident/never groups at baseline (30.8% vs. 0.0% / 6.1%, P<0.01).	Drop-outs had higher dental fear level. A tendency to maintain fear over time was predicted by psychiatric impairment and the personality factor neuroticism at baseline. Remission was predicted by personality factor extraversion. During the follow-up, dental anxiety increased / decreased in concert with the number of other fears.
Locker [11] *et al*. 1997	Randomly selected adult population in Etobicoke, Canada, n=1420. Subjects scoring ≥13 on the DAS or ≥8 on the Gatchel Fear Scale or reported being very afraid of the dental treatment were considered to be dentally anxious.	To study 1) the overlap between dental anxiety and BI (blood and injury) fears 2) the psychological characteristics of dentally anxious patients with and without BI fears 3) the contribution to BI fears make to dental anxiety	● DAS ● The Gatchel Fear Scale^48^ ● MQ (blood and injury fears) ● FSS-II (also other tests)	● Correlations between scores on MQ (measuring BI fears) and measures of dental anxiety were significant, but low (from 0.23 to 0.29, p<0.001 in all cases). ● Patients with dental anxiety had a mean MQ score of 4.87 (SD=3.23), and non-anxious patients a mean MQ score of 3.49 (SD=2.79; P<0.001). ● 16.1% of the patients with dental anxiety had BI fears.	BI phobics and dentally anxious patients without BI fears appeared to be rather similar in psychological characteristics. Agoraphobic symptoms, MQ score and number of psychiatric symptoms (and two other factors) had significant independent effects on DAS scores (F=80.15; P<0.001).	
Locker [2, 3] *et al*. 2001a, b	A birth cohort study, Dunedin, New Zealand. a) Assessment was undertaken at the age of 18 years, n=805. Subjects scoring ≥13 on the DAS (max. 20 pts.) were considered dentally anxious. Psychological health in the previous 12 months was measured using DIS. b) Longitudinal study, patients were examined at ages of 18 and 26 yrs, n=784. Incident cases were participants with DAS scores ≤12 at age 18 but a score of ≥13 at age 26. Dental visit and treatment experiences were examined.	a) Compare the prevalence of psychological disorders among dentally anxious and non-anxious groups b) Assess risk factors for the development of dental anxiety.	● DAS ● DIS	● Non-anxious vs. Moderately anxious *vs.* Severely anxious groups: - goraphobia 4.4 vs. 4.8 vs.13.9% - Social phobia 12.1 vs. 15.9 *vs.* 30.6% - Simple phobia 6.4 vs. 9.5 vs. 19.4% ● The difference was not significant concerning fear of blood, heights, storms, enclosed spaces, being in water, animals and insects. ● In a longitudinal study, incident cases had significantly higher scores than non-incident cases for simple phobia (p<0.001)	● Non-anxious vs. Moderately anxious vs. Severely anxious groups: Conduct disorder 6.9% vs. 14.3% vs. 19.4% ● One or more anxiety disorders: 21.6% vs. 27.0% vs. 52.8%. ● One or more anxiety OR one or more mood disorders: 30.3% vs. 39.7% vs. 58.3%. ● One or more anxiety and one or more mood disorders: 7.9% vs. 12.7% vs. 19.4%. In a longitudinal study, incident cases had significantly higher scores for major depressive episode ((P<0.05), generalized anxiety (P<0.01) and substance dependence (P<0.05).	The prevalence of comorbid phobias was largely accounted for by highly dentally anxious patients.
Moore *et al*.1991, Moore [31] *et al*. 1995	Dental Phobia Research and Treatment Center patients, Denmark, n=155. Additional subsample of patients with DAS score ≥15 was selected, n=80. Reference group of routine dental patients, same institution, n=148.	To explore the manifestations and acquisition of dental fear and to clarify diagnostic categories	● DAS ● STAI ● Modified FSS-II Geer Fear Scale59 ● DBS ● **Semi-structured interviews** were conducted for a subsample of 80 patients		The mean scores between test and reference group differed significantly between DAS and STAI-state (P<0.001) and between DAS and GFS (P<0.001)	

**Abbreviations** **BAI:** Beck's Anxiety Inventory [47] **BDI:** Beck's Depression Inventory [48] **DAS:** Dental Anxiety Scale [25] **DBS:** Dental Beliefs Survey [26] **DIS:** Diagnostc Interview Schedule [49] **DQ:** Disgust Questionnaire [50] **FSS-II:** Fear Survey Schedule II [51] **FQ-BI:** the Blood-injury Scale of the Fear Questionnaire [52] **MDAS:** The Modified Dental Anxiety Scale [29] **MQ:** Mutilation questionnaire [53] **SLC-90:** Revised Symptom Checklist [54] **SLC-90R:** (The Dutch version of) the Revised Symptom Checklist **STAI:** State-Trait anxiety inventory [55].

**Table 1b:** Dental anxiety and comorbid psychiatric symptoms and disorders. No control groups.

Authors	Study Population	Aim of the Study	Methods, Used Tests	Comorbid Phobias	Comorbid Psychiatric Symptoms	Comments
Berggren [38] 1992, Berggren [39] *et al*. 1995	Adult patients of Dental Fear Research and Treatment Centre, University of Gothenburg, Sweden, n=109	To investigate the presence, levels and relationships between general fears and specific dental fears.	● DAS ● DFS ● FSS-II and some additional items (phobias)	The correlation was significant between - DAS and FSS-II (r_p_=0.35) - DAS and GFS (r_p_=0.42) - DFS and FSS-II (r_p_=0.37) - DFS and GFS (r_p_=0.41)		
Hakeberg [36] *et al*. 2001	Patients of Dental Fear Research and Treatment Clinic, Gothenburg, Sweden. Adult patients, n=220	To explore the structural relationship between dental anxiety, mood, and general anxiety symptoms	● DAS ● DFS ● DBS ● STAI-S, STAI-T and GFS (anxiety) ● MACL (mood)		The association was significant between severe dental anxiety and mood (β=0.46), and between severe dental anxiety and general anxiety (β=0.17).	The study used the Structural Equation Modeling (SEM)^53,54^ approach.
Kaakko [35] *et al*. 1998	Employees of University of Washington, USA, n=350. Participants scoring ≥13 in the DAS were considered to be dentally anxious	To determine the extent and nature of fears in the population	● DAS ● Four six-point questions adapted from SF-36 Health Survey [55] (Mental health score, anxiety and depression-related symptoms).		● Fearful respondents reported high proportion of anxiety and depression. ● The correlation between mental health score and DAS was significant (r=0.17, p=0.006).	
Moore [31] *et al*.1995	Dental Phobia Research and treatment center patients, Denmark, n=80	To examine dentally phobic patients by psychometric testing and clinical interviews	● STAI-T ● GFS (modified) ● MACL ● **Clinical interviews**	● Social phobia 46% ● Specific phobia 19% ● Multiple phobia 28%	GAD (generalised anxiety disorder) 38%	
Pekkan [37] *et al*. 2011	Patients of Dumlupinar University Hospital Dental Clinic, Turkey, n=250	To investigate the relationship between dental anxiety, depression, and general anxiety level and their differences among genders	● MDAS ● BAI ● BDI		● The correlation was significant between mean scores of MDAS and BDI (r=0.148, p=0.019) and BAI mean scores (r=0.273, p<0.01). ● When the cut-off point of the MDAS was taken as 19, there was a significant correlation between MDAS and BAI, but not with BDI ● When the cut-off point was taken as 15, there was significant correlation between MDAS and both BAI and BDI.	19 pts (out of maximum of 25 pts) or more in the MDAS test is widely considered to mean that a patient is dentally anxious.
Roy-Byrne [12] *et al*. 1994	Patients of University of Washington Dental Fears Research clinic, USA, n=73. Patients were divided into different groups	To study diagnostic and psychopathological characters of subjects with dental phobia	● DFS ● **Clinical interviews**	● 60% of the patients had current simple phobia as the only diagnosis. ● 45% of the patients had at least one additional simple phobia BESIDES dental phobia: - flying 20% - heights 20% - enclosed spaces 10% - animals 10% - blood 4% - any 45%	● 29 of the 73 patients had additional current DSM-III Axis I diagnosis: -anxiety 20% -mood 16% -substance abuse 4% ● A total of 68% of patients had lifetime (current & past) Axis I diagnosis. ● 61% had at least one Axis II personality disorder and 35% more than one diagnosis.	In patients with simple phobia, the subgroup of patients with additional current Axis I diagnosis had a higher proportion of Axis II diagnoses (86% vs. 45%).
Tellez [13] *et al*. 2015	Patients scheduled for dental appointment at Temple University dental clinic, Philadelphia, USA, n=120	To examine the association between dental anxiety and pain and other psychological variables	● Anxiety Disorders Interview Schedule DSM-IV^56^ ● MDAS ● the 5-iten subscale of FQ-BII^57^ ● SAAS	Dental anxiety was positively correlated with BII fears (r=0.47, P<0.001).	Dental anxiety was positively correlated with social appearance anxiety (r=0.39, P<0.001).	Dental anxiety was positively correlated with pain experienced at the last dental appointment (P<0.001).

**Abbreviations** **BAI:** Beck’s Anxiety Inventory [47] **BDI:** Beck’s Depression Inventory [48] **DAS:** Dental Anxiety Scale [25] **DBS:** Dental Beliefs Survey [26] **DFS:** Dental Fear Survey [27] **FSS-II:** Fear Survey Schedule II [51] **FQ-BII:** the Blood-injury Scale of the Fear Questionnaire [52] **GFS:** Geer Fear Scale [56] **MACL:** Mood Adjective Checklist [57] **MDAS:** The Modified Dental Anxiety Scale [29] **SAAS:** Social Appearance Anxiety Scale [58] **STAI:** State-Trait anxiety inventory [55].

**Table 2:** Summary of results. The findings were classified as diagnoses if clinical interviews were used in the original study, and, if only tests were used, the findings were considered as symptoms (note: In Tellez 2015 a semi-structured clinical interview was administered by telephone, and the findings were considered as symptoms).

**Comorbid Findings and Studies with Statistically Significant Associations**	**Diagnoses in Population-Based Studies**	**Symptoms in Population-Based Studies**	**Diagnoses in patient-Based Studies**	**Symptoms in Patient-Based Studies**
**Anxiety-related disorders** 9 studies with positive correlations	**(Hägglin *et al.* 2001)	*(Kaakko *et al.* 1998) ***(Halonen *et al.* 2014, Locker *et al.* 2001)	Roy-Byrne *et al.* 1994: 20%^1^ Moore *et al.* 1995: 30% ^1^ (GAD)	*(Hakeberg *et al.* 2001, Pekkan *et al.* 2011) ***(Aartman *et al.* 1997, DeJongh *et al.* 1998)
**Agoraphobia** 3 studies with positive correlations	(no studies)	*(Locker *et al.* 2001) ***(Locker *et al.* 1997)	(no studies)	**(Aartman *et al.* 1997)
**Social phobia** 3 studies with positive correlations	(no studies)	**(Locker *et al.* 2001)	Moore *et al.* 1995: 46% ^1^	**(Tellez 2015)
**Specific (simple) phobia (other than dental phobia)** 7 studies with positive correlations	***(Hägglin *et al.* 2001)	*(Locker *et al.* 2001) ***(Locker *et al.* 1997)	Moore *et al.* 1995: 19% ^1^ Roy-Byrne *et al.* 1994: 45% ^1^	***(Tellez 2015, DeJongh *et al.* 1998)
**Mood disorder/ depression** 8 studies with positive correlations	**(Hägglin *et al.* 2001)	***(Halonen *et al.* 2014) *(Kaakko *et al.* 1998, Locker *et al.* 2001)	Roy-Byrne *et al.* 1994: 16% ^1^	*(Pekkan *et al.* 2011) **(Aartman *et al.* 1997, Hakeberg *et al.* 2001)
**Substance & alcohol use disorder** 2 studies with positive correlations	(no studies)	*(Locker *et al.* 2001)	Roy-Byrne *et al.* 1994: 4% ^1^	(no studies)
**Other DSM-IV Axis I disorders** 4 studies with positive correlations	**psychiatric impairment and ***social disability (Hägglin *et al.* 2001)	***number of other psychiatric symptoms (Locker *et al.* 1997) *conduct disorder (Locker *et al.* 2001)	(no studies)	*somatization *cognitive-performance difficulty **interpersonal sensitivity and paranoid ideation (Aartman *et al.* 1997)

Cohen's d <0.5 **or** Pearson's r <0.3 **or** Odds Ratio <2.5 **or** Difference in percentage <18 ** Cohen's d<0.8 **or** Pearson's r <0.5 **or** Odds Ratio <4 **or** Difference in percentage <30 *** Cohen's d ≥0.8 **or** Pearson's r ≥0.5 **or** Odds Ratio ≥4 **or** Difference in percentage ≥30 (Rosenthal 1996) **^1^** Prevalence of comorbid diagnosis in dentally anxious participants.

**Appendix Table:** Description of the tests used to measure dental anxiety in reviewed articles. All tests are widely used in clinical and research practice.

**Test**	**No. of Items**	**Description of the Test**
Dental Anxiety Scale DAS [25]	4	Level of dental anxiety is measured from 4 (no fear) to 20 (extreme fear).
Dental Beliefs Survey DBS [26]	15	Explores patients’ confidence in dentist-patient interaction on a scale from 15 (highly positive beliefs) to 75 (highly negative beliefs).
Dental Fear Survey DFS [27]	20	Test varies from 20 to 100 and assesses 3 different areas of fear reactions: avoidance, autonomic arousal and fear of specific objects or situations.
Gatchel Fear Scale GFS [28]		Subjects are asked to rate their fear of dentist on a scale in which 1 means no fear, 5 moderate and 10 extreme fear.
Modified Dental Anxiety Scale MDAS [29]	5	Level of dental anxiety is measured from 5 (no fear) to 25 (extreme fear).

## APPENDIX 1

Checklist For Measuring Study Quality

(If the answer to the question is YES, log it as 1 point, if NO, log it as 0 points).

**Reporting**

1. Is the hypothesis / aim / objective of the study clearly described?
2. Are the main outcomes to be measured clearly described in the Introduction or Methods section?
3. Are the characteristics of the patients included in the study clearly described?
4. Are the interventions of interest clearly described?
5. Are the main findings of the study clearly described?
6. Have all the important adverse events that may be a consequence of the intervention been reported?

**External Validity**

1. Were the subjects asked to participate in the study representative of the entire population from which they were recruited?

**Internal Validity - Bias**

1. Were the statistical tests used to assess the main outcomes appropriate?
2. Were the main outcome measures used accurate (valid and reliable)?

**Internal Validity – Confounding (Selection Bias)**

1. Were the patients in different intervention groups (trials and cohort studies) or were the cases and controls (case-control studies) recruited from the same population?
2. Was there adequate adjustment for confounding in the analyses from which the main findings were drawn?
3. Were losses of patients to follow-up taken into account?

## References

[1] Armfield J.M. (2010). The extent and nature of dental fear and phobia in Australia.. Aust. Dent. J..

[2] Locker D., Poulton R., Thomson W.M. (2001). Psychological disorders and dental anxiety in a young adult population.. Community Dent. Oral Epidemiol..

[3] Locker D., Thomson W.M., Poulton R. (2001). Psychological disorder, conditioning experiences, and the onset of dental anxiety in early adulthood.. J. Dent. Res..

[4] Smith T.A., Heaton L.J. (2003). Fear of dental care: Are we making any progress?. J. Am. Dent. Assoc..

[5] Locker D., Shapiro D., Liddell A. (1996). Who is dentally anxious? Concordance between measures of dental anxiety.. Community Dent. Oral Epidemiol..

[6] Heidari E., Banerjee A., Newton J.T. (2015). Oral health status of non-phobic and dentally phobic individuals; A secondary analysis of the 2009 adult dental health survey.. Br. Dent. J..

[7] Nicolas E., Collado V., Faulks D., Bullier B., Hennequin M. (2007). A national cross-sectional survey of dental anxiety in the French adult population.. BMC Oral Health.

[8] Chanpong B., Haas D.A., Locker D. (2005). Need and demand for sedation or general anesthesia in dentistry: A national survey of the canadian population.. Anesth. Prog..

[9] World Health Organization (1992). The ICD-10 classification of mental and behavioral disorders. Clinical descriptions and diagnostic guidelines..

[10] American Psychiatric Association (1994). Diagnostic and statistical manual of mental disorders (DSM-IV)..

[11] Locker D., Shapiro D., Liddell A. (1997). Overlap between dental anxiety and blood-injury fears: Psychological characteristics and response to dental treatment.. Behav. Res. Ther..

[12] Roy-Byrne P., Milgrom P., Katon W. (1994). Psychopathology and psychiatric diagnosis in subjects with dental phobia.. J. Anx. Dis..

[13] Tellez M., Kinner D.G., Heimberg R.G., Lim S., Ismail A.I. (2015). Prevalence and correlates of dental anxiety in patients seeking dental care.. Community Dent. Oral Epidemiol..

[14] Schatzberg A.F., Samson J.A., Rothschild A.J., Bond T.C., Regier D.A. (1998). McLean hospital depression research facility: Earlyonset phobic disorders and adult-onset major depression.. Br. J. Psychiatry.

[15] Choy Y., Fyer A.J., Goodwin R.D. (2007). Specific phobia and comorbid depression: A closer look at the national comorbidity survey data.. Compr. Psychiatry.

[16] Trumpf J., Margraf J., Vriends N., Meyer A.H., Becker E.S. (2010). Specific phobia predicts psychopathology in young women.. Soc. Psychiatry Psychiatr. Epidemiol..

[17] Kaufman J., Charney D. (2000). Comorbidity of mood and anxiety disorders.. Depress. Anxiety.

[18] Boschloo L., Vogelzangs N., Smit J.H., van den Brink W., Veltman D.J., Beekman A.T., Penninx B.W. (2011). Comorbidity and risk indicators for alcohol use disorders among persons with anxiety and/or depressive disorders: Findings from the netherlands study of depression and anxiety (NESDA).. J. Affect. Disord..

[19] de Graaf R., ten Have M., Tuithof M., van Dorsselaer S. (2013). First-incidence of DSM-IV mood, anxiety and substance use disorders and its determinants: Results from the netherlands mental health survey and incidence study-2.. J. Affect. Disord..

[20] Baldwin D.S., Evans D.L., Hirschfeld R.M., Kasper S. (2002). Can we distinguish anxiety from depression?. Psychopharmacol. Bull..

[21] Stroup D.F., Berlin J.A., Morton S.C., Olkin I., Williamson G.D., Rennie D., Moher D., Becker B.J., Sipe T.A., Thacker S.B. (2000). Meta-analysis of observational studies in epidemiology: A proposal for reporting. Meta-Analysis Of Observational Studies in Epidemiology (MOOSE) group.. JAMA.

[22] Downs S.H., Black N. (1998). The feasibility of creating a checklist for the assessment of the methodological quality both of randomised and non-randomised studies of health care interventions.. J. Epidemiol. Community Health.

[23] American psychiatric association (1980). Diagnostic and statistical manual of mental disorders (DSM-III)..

[24] Rosenthal J.A. (1996). Qualitative descriptors of strength of association and effect size.. J. Soc. Serv. Res..

[25] Corah N.L. (1969). Development of a dental anxiety scale.. J. Dent. Res..

[26] Milgrom P., Weinstein P., Kleinknecht R.A., Getz T. (1985). Treating fearful dental patients: A clinical handbook..

[27] Kleinknecht R.A., Klepac R.K., Alexander L.D. (1973). Origins and characteristics of fear of dentistry.. J. Am. Dent. Assoc..

[28] Gatchel R.J. (1989). The prevalence of dental fear and avoidance: Expanded adult and recent adolescent surveys.. J. Am. Dent. Assoc..

[29] Freeman R., Clarke H.M.M., Humphris G.M. (2007). Conversion tables for the corah and modified dental anxiety scales.. Community Dent. Health.

[30] Aartman I.H.A., de Jongh A., van der Meulen M.J. (1997). Psychological characteristics of patients applying for treatment in a dental fear clinic.. Eur. J. Oral Sci..

[31] Moore R., Brødsgaard I. (1995). Differential diagnosis of odontophobic patients using the DSM-IV.. Eur. J. Oral Sci..

[32] De Jongh A., Bongaarts G., Vermeule I., Visser K., De Vos P., Makkes P. (1998). Blood-injury-injection phobia and dental phobia.. Behav. Res. Ther..

[33] Hägglin C., Hakeberg M., Hällström T., Berggren U., Larsson L., Waern M., Pálsson S., Skoog I. (2001). Dental anxiety in relation to mental health and personality factors. A longitudinal study of middle-aged and elderly women.. Eur. J. Oral Sci..

[34] Halonen H., Salo T., Hakko H., Räsänen P. (2014). The association between dental anxiety, general clinical anxiety and depression among finnish university students.. Oral Health Dent. Manag..

[35] Kaakko T., Milgrom P., Coldwell S.E., Getz T., Weinstein P., Ramsay D.S. (1998). Dental fear among university employees: Implications for dental education.. J. Dent. Educ..

[36] Hakeberg M., Hägglin C., Berggren U., Carlsson S.G. (2001). Structural relationships of dental anxiety, mood, and general anxiety.. Acta Odontol. Scand..

[37] Pekkan G., Kilicoglu A., Hatipoglu H. (2011). Relationship between dental anxiety, general anxiety level and depression in patients attending a university hospital dental clinic in Turkey.. Community Dent. Health.

[38] Berggren U. (1992). General and specific fears in referred and self-referred adult patients with extreme dental anxiety.. Behav. Res. Ther..

[39] Berggren U., Carlsson S.G., Gustafsson J-E., Hakeberg M. (1995). Factor analysis and reduction of a fear survey schedule among dental phobic patients.. Eur. J. Oral Sci..

[40] Halonen H., Salo T., Hakko H., Räsänen P. (2012). Association of dental anxiety to personality traits in a general population sample of Finnish University students.. Acta Odontol. Scand..

[41] Lautch H. (1971). Dental phobia.. Br. J. Psychiatry.

[42] Ray J., Boman U.W., Bodin L., Berggren U., Lichtenstein P., Broberg A.G. (2010). Heritability of dental fear.. J. Dent. Res..

[43] Vassend O., Røysamb E., Nielsen C.S. (2011). Dental anxiety in relation to neuroticism and pain sensitivity. A twin study.. J. Anxiety Disord..

[44] Jacobi F., Höfler M., Siegert J., Mack S., Gerschler A., Scholl L., Busch M.A., Hapke U., Maske U., Seiffert I., Gaebel W., Maier W., Wagner M., Zielasek J., Wittchen H.U. (2014). Twelve-month prevalence, comorbidity and correlates of mental disorders in Germany: The mental health module of the german health interview and examination survey for adults (DEGS1-MH).. Int. J. Methods Psychiatr. Res..

[45] Kessler R.C., Chiu W.T., Demler O., Merikangas K.R., Walters E.E. (2005). Prevalence, severity, and comorbidity of 12-month DSM-IV disorders in the national comorbidity survey replication.. Arch. Gen. Psychiatry.

[46] Slade T., Johnston A., Oakley Browne M.A., Andrews G., Whiteford H. (2009). 2007 national survey of mental health and wellbeing: Methods and key findings.. Aust. N. Z. J. Psychiatry.

[47] Beck A.T., Epstein N., Brown G., Steer R.A. (1988). An inventory for measuring clinical anxiety: Psychometric properties.. J. Consult. Clin. Psychol..

[48] Beck A.T., Ward C.H., Mendelson M., Mock J., Erbaugh J. (1961). An inventory for measuring depression.. Arch. Gen. Psychiatry.

[49] Robins L.N., Helzer J.E., Croughan J., Ratcliff K.S. (1981). National institute of mental health diagnostic interview schedule. Its history, characteristics, and validity.. Arch. Gen. Psychiatry.

[50] Rozin B., Fallon A.E., Mandell R. (1984). Family resemblance in attitude to foods.. Dev. Psychol..

[51] Geer J.H. (1965). The development of a scale to measure fear.. Behav. Res. Ther..

[52] Marks I.M., Mathews A.M. (1979). Brief standard self-rating for phobic patients.. Behav. Res. Ther..

[53] Klorman R., Hastings J., Weerts T., Melamed B., Lang P. (1974). Psychometric description of some specific-fear questionnaires.. Behav. Ther..

[54] Degoratis L.R. (1977). SLC-90: Administration, scoring and procedures manual -I for the (revised) version..

[55] Spielberg C.D., Gorsuch R.C., Lushene R.E. (1970). Manual for the state-trait-anxiety inventory..

[56] Geer J.H. (1965). The development of a scale to measure fear.. Behav. Res. Ther..

[57] Sjöberg L., Svensson E., Persson L-O. (1979). The measurement of mood.. Scand. J. Psychol..

[58] Hart T.A., Flora D.B., Palyo S.A., Fresco D.M., Holle C., Heimberg R.G. (2008). Development and examination of the social appearance anxiety scale.. Assessment.

